# Recent Progress in *in vitro* Models for Atherosclerosis Studies

**DOI:** 10.3389/fcvm.2021.790529

**Published:** 2022-01-27

**Authors:** Jun Chen, Xixi Zhang, Reid Millican, Tyler Lynd, Manas Gangasani, Shubh Malhotra, Jennifer Sherwood, Patrick Taejoon Hwang, Younghye Cho, Brigitta C. Brott, Gangjian Qin, Hanjoong Jo, Young-sup Yoon, Ho-Wook Jun

**Affiliations:** ^1^Department of Biomedical Engineering, The University of Alabama at Birmingham, Birmingham, AL, United States; ^2^Endomimetics, LLC., Birmingham, AL, United States; ^3^Family Medicine Clinic, Obesity, Metabolism, and Nutrition Center and Research Institute of Convergence of Biomedical Science and Technology, Pusan National University Yangsan Hospital, Yangsan, South Korea; ^4^Division of Cardiovascular Disease, School of Medicine, The University of Alabama at Birmingham, Birmingham, AL, United States; ^5^Wallace H. Coulter Department of Biomedical Engineering, Georgia Institute of Technology and Emory University, Atlanta, GA, United States; ^6^Division of Cardiology, Department of Medicine, Emory University, Atlanta, GA, United States; ^7^Severance Biomedical Science Institute, Yonsei University College of Medicine, Seoul, South Korea

**Keywords:** atherosclerosis, disease models, tissue-engineered blood vessels, microfluidic chips, *in vitro* models and methods

## Abstract

Atherosclerosis is the primary cause of hardening and narrowing arteries, leading to cardiovascular disease accounting for the high mortality in the United States. For developing effective treatments for atherosclerosis, considerable efforts have been devoted to developing *in vitro* models. Compared to animal models, *in vitro* models can provide great opportunities to obtain data more efficiently, economically. Therefore, this review discusses the recent progress in *in vitro* models for atherosclerosis studies, including traditional two-dimensional (2D) systems cultured on the tissue culture plate, 2D cell sheets, and recently emerged microfluidic chip models with 2D culture. In addition, advanced *in vitro* three-dimensional models such as spheroids, cell-laden hydrogel constructs, tissue-engineered blood vessels, and vessel-on-a-chip will also be covered. Moreover, the functions of these models are also summarized along with model discussion. Lastly, the future perspectives of this field are discussed.

## Introduction

Cardiovascular disease (CVD) is the severest global health concern and the primary leading cause of mortality in the United States, resulting primarily from atherosclerosis (1, 2). *In vivo* models, ranging from rats to pigs to non-human primates, have been employed as the gold standard to explore valuable insights into atherosclerosis and predict novel drug safety and efficacy for atherosclerosis treatment (3, 4). However, current *in vivo* atherosclerosis models suffer issues, such as inevitable interspecies differences in the genome, biological varieties, limited genetic variability, and low throughput. In particular, large-sized *in vivo* atherosclerosis model development requires a costly high-fat diet, genetic manipulation, and extensively long induction time, leading to a prolonged life cycle of pharmaceutical research with increased expense (3). In addition, these issues in large animal models also have hindered the widespread use of these systems in research. In contrast, the induction of atherosclerosis models in small animals, such as mice and rabbits, is cheaper and less time consuming; however, these models commonly develop atherosclerotic lesions in carotid arteries and aortic arches, different from the patients who often develop atherosclerosis in their coronary arteries (3, 4). Given these unsolved issues, there is a high chance that the newly discovered lead compounds showing therapeutic efficacy in the animal models would fail to demonstrate comparable efficacy in patients. In comparison to *in vivo* models, *in vitro* models provide great opportunities to assess drug efficiency and toxicity as well as explore biological mechanisms in a reproducible, economical, high throughput, and controllable manner (5, 6). Those advantages render them valuable platforms for atherosclerosis-associated mechanistic investigations and new therapy development.

*In vitro* models are usually classified into 2D and 3D models. The traditional *in vitro* 2D models generally refer to the monolayer of cells created by seeding cells on the tissue culture plates (TCP) followed by cell culture in a static condition. In this system, the cells usually adhere and spread on the flat surface. Due to their high availability and low cost, such systems are the most widely used to evaluate drug efficacy and toxicity and study biological processes. In addition to the traditional 2D systems, cell sheet tissue engineering and microfluidic technology have brought about tissue-engineered 2D cell sheets and microfluidic chips with 2D culture to improve the recapitulation of the human physiological and pathological environment (7–9). However, despite being more advanced than traditional 2D systems, both models still cannot faithfully replicate the human pathophysiology, thereby encouraging more advanced *in vitro* models. An ideal *in vitro* system should emulate the 3D human tissue architecture with proper cellular components and disease features. Therefore, a considerable effort has been made to develop *in vitro* 3D models with structures that mimic human 3D tissues for modeling pathological processes for elucidating disease development and new drug evaluation, ranging from cancer (10), the blood-brain barrier (11, 12), neurodegenerative diseases (13), cardiac fibrosis (14), to atherosclerosis (15).

**Figure 1:**
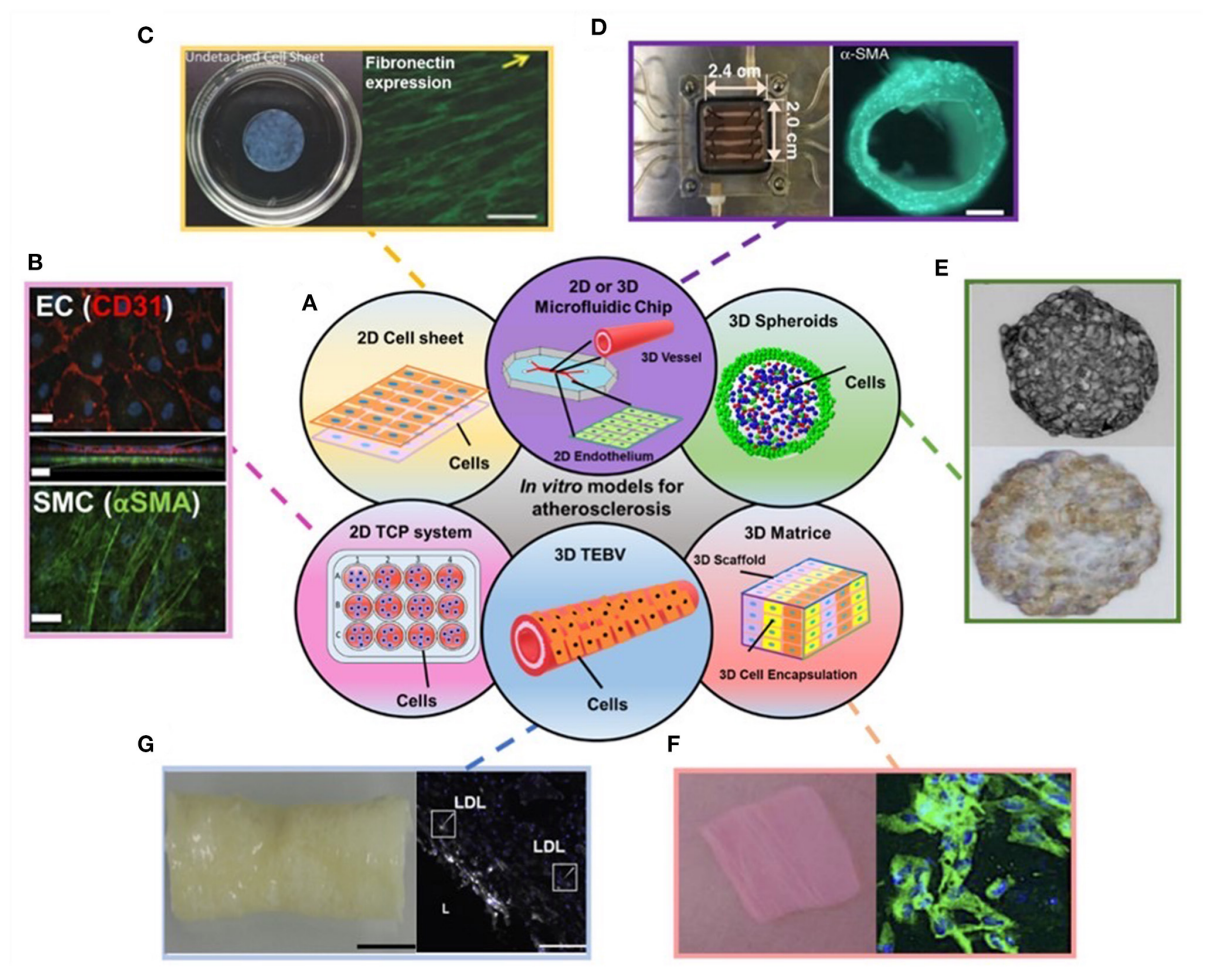
**(A)** Schematic showing the disused *in vitro* models for atherosclerosis studies in the review. **(B–G)** Examples of data regarding different *in vitro* models covered in the review: **(B)** Endothelial cells (ECs) (top) and Smooth muscle cells (SMCs) (bottom) stained with EC and SMC phenotype markers in 2D transwell model. **(C)** 2D cell sheet (left) that expresses fibronectin (right). **(D)** 3D microfluidic vessel on a chip (left) made of green fluorescence protein-expressing human umbilical vein endothelial cells (right). **(E)** SMC spheroid (top) and EC/SMC spheroid (bottom). **(F)** 3D SMC laden hydrogel constructs (left) and stained with SMA-α (right, green). **(G)** 3D tissue-engineered blood vessels (left) and stained with monocytes and LDL (right). Adapted, with permission from (16) **(B)**, (17) **(C)**, (18) **(D)**, (19) **(E)**, (20) **(F)**, and (21) **(G)**.

In this review, we summarize the recent progress in *in vitro* models for atherosclerosis studies, including 2D *in vitro* models, such as traditional 2D cultures on TCP, 2D tissue-engineered cell sheets, and newly emerging microfluidic chip models with 2D culture. Moreover, we discuss the state-of-the-art *in vitro* three-dimensional (3D) models in the context of spheroids, cell-laden hydrogel constructs, tissue-engineered blood vessels (TEBVs), and vessel-on-a-chips (Figure 1). Pathology of atherosclerosis is also included to facilitate understanding of these models and their applications for mechanistic studies. Lastly, we also highlight the future perspectives of this field.

## Pathology of Atherosclerosis

**Figure 2:**
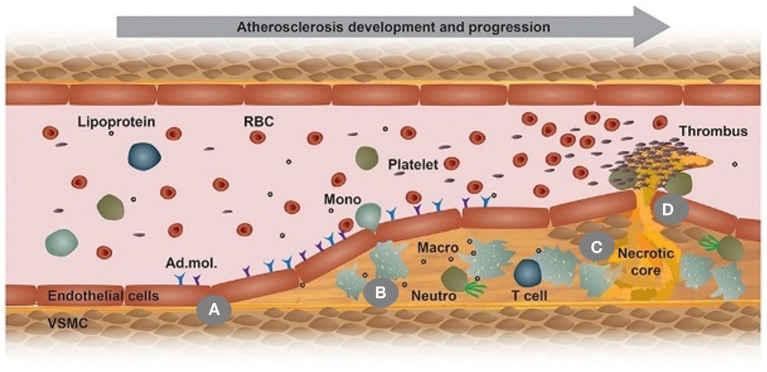
This scheme illustrates the development of an atherosclerotic plaque from left to right in a longitudinal section of an arterial vessel: **(A)** Upon activation by metabolic or inflammatory triggers, endothelial cells express adhesion molecules (Ad. mol.) that promote the recruitment of monocytes (Mono); **(B)** Monocyte differentiate into macrophages (Macro) and uptake Ox-LDL, leading to foam cell formation; **(C)** Macrophage (Macro) and foam cells eventually die and fall apart, thereby forming a necrotic core; **(D)** Advanced, vulnerable plaques can rupture and thereby form an arterial thrombus. Adapted, with permission, from (23) **(A–D)**.

Atherosclerosis, a chronic inflammatory disease, results in plaque formation within the intimal layer of arteries (22). Several risk factors can increase the likelihood of atherosclerosis development. The most well-known is low-density lipoprotein cholesterol (LDL-C). Typically, atherosclerosis is initiated by the passage of LDL-C through arterial endothelium and accumulates within the intima, which induces endothelial dysfunction with adhesion molecule expression, an essentially biological process for atherosclerosis initiation (Figure 2A) (23). Similar to LDL-C, an abnormal and turbulent flow within the arterial lumen also elevates the risk of atherosclerosis by inducing adhesive molecule expression (24). High-density lipoprotein cholesterol (HDL-C) is inversely related to the risk of developing atherosclerosis; however, evidence suggests no protective role for HDL-C against atherosclerosis (25). The seemingly futile behavior could be due to compositional and functional modifications of HDL-C by inflammatory mediators (26). Other risk factors may include hypertension, chronic kidney disease, aging, and hyperglycemia (27).

Although there is controversy, the most prevalent theory describes the progression of atherosclerosis strongly depending on the activation of the inflammatory response; thus, circulating monocytes, critical players within innate immunity playing a crucial role in inflammation activation. Specifically, those monocytes are responsible for infiltrating the lesion site, differentiating into macrophages, and internalizing oxidized LDL (Ox-LDL) that gives rise to foam cells, the primary cellular components found in the plaque, thereby triggering an inflammatory reaction (Figure 2B) (23, 28). Additionally, some evidence suggests that macrophages may locally proliferate to accelerate foam cell genesis (29). As the disease progresses, a fatty streak composed of aggregated foam cells forms in the lesion and further aggravates the inflammation. As the disease advances by increased inflammation, an atheroma may grow in the lesion and becomes increasingly fibrous. With the inflammation continuously becoming severe, local cells undergo apoptosis. The intense inflammatory response in the plaque may be mitigated if efferocytosis occurs, whereby macrophages clear apoptotic cells; however, in most cases, as the atherosclerotic plaque advances, macrophages experience cellular reprogramming that restrains their efferocytotic capacity. As more and more apoptotic cells fail to be removed, a necrotic core forms within the lesion (Figure 2C) (23, 30).

In addition to monocytes and macrophages, smooth muscle cells (SMCs) play a vital but paradoxical role in atherosclerosis by either advancing or protecting disease progression. Since SMCs are not terminally differentiated, they undergo phenotype transition in an atherosclerotic environment. The phenotype variations of SMCs may implicate a loss of contractility, reduced SMC contractile markers, increased proliferation and migration of SMCs (31), and upregulated production of proteoglycans. It is worth noting that SMCs can express macrophage and endothelial cell markers instigating ambiguity among the atherosclerotic players. Additionally, recent studies showed that SMCs could transform into macrophage-like cells, internalize Ox-LDL, and become foam cells (31, 32), thus contributing to the generation of the necrotic core and promoting atherosclerosis progression. Despite that, SMC phenotypes can also produce a fibrous cap that stabilizes the plaque, reduces the risk of rupture, and prevents thrombus formation. Without such stabilization, the plaque may rupture, leading to thrombosis that often occurs in advanced stages of atherosclerosis (Figure 2D) (23). In addition, SMCs present within the atheromatous plaque may form a mineralized matrix resulting in calcium deposits. Early microcalcifications have been shown to destabilize the plaque, while more extensive calcifications serve as a stabilizing component to the atheroma (33, 34).

## *In vitro* 2D Models for Atherosclerosis Studies

2D *in vitro* models are essential for studying the pathology of various diseases and drug evaluation, which have been extensively used for decades. The most common 2D *in vitro* models are single-cell culture systems, which contain only one type of cell component observed in the atherosclerotic plaque, such as ECs, SMCs, macrophages, and foam cells. Single-cell cultures have been used to assess new types of therapeutics, such as microRNA (35, 36) and exosomes (37, 38), and investigate mechanistic studies associated with atherosclerosis. However, recently they have been widely applied for evaluating the efficacy of drug-loaded delivery systems for treating atherosclerosis due to the issues observed in free drug administration (Table 1). Despite the wide use of single-cell models, single-cell models may not be sufficient to obtain a reliable prediction for therapeutic efficacy in patients due to their inability to mimic the native structure of vessels and features of human plaques. Thus, to gain significant insights into therapeutic efficacy and atherosclerosis pathogenesis, single-cell models are commonly used along with *in vivo* models.

**Table 1:** Summary of *in vitro* 2D single-cell culture model and its specific function for evaluating drug delivery system for atherosclerosis.

**Model**	**Model function (biological process studied)**	**References**
Macrophage	To evaluate drug delivery system targeting ability to plaque	(39)
	To evaluate drug delivery system effects on macrophage polarization	(40)
	To evaluate drug delivery system effects on cholesterol removal (efflux)	(41–43)
	To evaluate drug delivery system effects on inflammation resolution	(44–50)
	To evaluate drug delivery system effects on Reactive oxygen species generation	(41, 47, 49, 51–54)
	To evaluate drug delivery system effects on nitric oxide production	(52)
	To evaluate drug delivery system effects on efferocytosis and phagocytosis	(55, 56)
	To evaluate cellular uptake of drug delivery system	(41, 43, 44, 49, 55, 56)
	To evaluate drug delivery system effects on cellular apoptosis	(49, 57)
	To evaluate drug delivery system effects on foam cell formation	(41, 42, 46, 49, 57)
SMC	To evaluate drug delivery system effects on the cellular proliferation	(44)
	To evaluate drug delivery system effects on foam cell formation	(49, 58)
	To evaluate the cellular uptake of drug delivery systems	(49)
EC	To evaluate the cellular uptake of drug delivery systems	(44)
Foam cell	To evaluate cellular binding of drug delivery systems	(51)
	To evaluate drug delivery system effects on cholesterol removal (efflux)	(54)
	To evaluate drug delivery system effects on inflammation resolution	(54)

In addition to the single-cell culture systems, research has been focused on developing co-culture models for atherosclerosis-associated studies. The co-culture systems include direct cell-to-cell and indirect transwell co-cultures. It was reported that, as far back as 1986, various cell types involved in atherosclerosis pathology—including ECs, SMCs, and macrophages—were used in various direct and indirect co-culture studies to create blood vessels for examining inflammation and its role in atherosclerosis (59–63). Furthermore, with the advancement of the engineering approach, considerable efforts have been made to develop advanced *in vitro* 2D systems, where cells are seeded in 2D scaffolds or microfluidic chips for better mimicry of the physiology and pathology of atherosclerosis environment. In this section, we summarize these systems.

## Single-Cell Model

Single-cell models have been widely used for assessing drug delivery systems due to their high availability and ease of fabrication. These single systems include only macrophages, SMCs, ECs, or foam cells (Table 1). One of the applications of these single-cell systems in the drug delivery field is to evaluate the cellular uptake of those systems. In one study, Schwendeman et al. developed a single-cell model composed of THP-1 monocytes differentiated macrophages to evaluate the cellular uptake of these DiD dye-labeled synthetic high-density lipoproteins (DiD-sHDL) (41). It was shown that 99% of macrophages showed DiD positive after 2 h incubation with the particles. In addition to macrophages, other cell types, such as TNF-α treated HUVECs and mouse vascular smooth muscle cells (VSMCs), have also been employed to demonstrate the efficient cellular uptake of DiD labeled macrophage membrane functionalized biomimetic nanoparticles and Cy5 labeled β-cyclodextrin nanoparticles, respectively (44). Besides, the Liu group reported the development of single-cell systems composed of either HUVECs, RAW cells, or foam cells to investigate the cellular binding of platelet mimicking nanoparticles to these cells. Using these single-cell systems, they found those platelet mimicking nanoparticles showed strong binding to foam cells in contrast to no binding to other types of cells (51).

The anti-atherosclerotic function of a therapeutic loaded drug delivery system is dependent on the effects of these drug delivery systems on several critical biological processes strongly associated with macrophages or macrophage-derived foam cells, such as ROS generation, foam cell formation, and cholesterol efflux, and inflammation resolution (Table 1). Thus, macrophages have been widely used to evaluate the drug delivery system's effect on these processes. One typical example is using the macrophages to evaluate the regulation of the cholesterol efflux and influx resulting from drug delivery systems by Ghosh et al. They showed that the simultaneous delivery of siRNA and LXR ligand *via* mannose-functionalized dendrimeric nanoparticles (mDNPs) could significantly decrease cholesterol content in the macrophages (42). In addition, macrophages in the plaque have been demonstrated to express ca^2+/^calmodulin-dependent solid protein kinase (CaMKIIγ) and suppress the efferocytosis receptor, thereby leading to plaque necrosis and efferocytosis suppression. Macrophage systems have been used to investigate the drug delivery system's effect on efferocytosis and plaque stability. For checking the effect of siRNA nanoparticles on efferocytosis, bone marrow-derived macrophages were incubated with siRNA-loaded nanoparticles by the Shi group. It was shown that the CaMKIIγ expression was significantly decreased by the siRNA-loaded nanoparticles, thus increasing macrophage efferocytosis and plaque stability (55). Besides enhancing efferocytosis, the induction of inflammation resolution by therapeutic-loaded drug delivery systems is also investigated for treating atherosclerosis. One typical example demonstrated by the Scott group, where the macrophages were treated with the anti-inflammatory nanocarriers, celastrol-loaded nanocarriers, showed significantly less production of TNF-α than those untreated ones (45).

In addition to evaluating therapeutic loaded drug delivery systems, single-cell models have been applied to explore the mechanism associated with atherosclerosis pathogenesis. For instance, ECs have been used to evaluate various pathophysiological stimuli, such as pro-inflammatory cytokines, hemodynamic forces, hypercholesterolemia, and hypertension, on endothelial dysfunction, inflammation, and cellular senescence in atherogenesis (24, 64–70). In addition, Single-cell systems composed of SMCs or monocytes have been used to assess these stimuli' effects on SMC phenotype change, monocyte differentiation, and foam cell formation, which may be a critical process in developing atherosclerosis (71–80). Table 2 summarizes some recent typical single-cell model examples of atherosclerosis mechanism exploration.

**Table 2:** Some examples in recent studies using a single-cell model for atherosclerosis mechanism exploration.

**Model**	**Biological process**	**Model function**	**References**
Macrophage	Efferocytosis or/and phagocytosis, or pyroptosis	To study the effect of allele G of rS9349379, an intron of PHACTR1 gene, on impairing the efferocytosis in human atherosclerotic lesional macrophages	(81)
		To explore whether GATA2 overexpression can impair macrophage phagocytosis and efferocytosis	(82)
		To study the mitochondrial outer membrane protein effect on inhibiting macrophage pyroptosis resulting from Ox-LDL	(83)
	Cellular senescence	To explore the mechanism of formation of senescent macrophages during atherosclerosis and whether LPS can induce macrophage senescence	(84)
	Lipid uptake or/and foam cell formation	To investigate the direct role of IgE on macrophage-sterol-responsive-network gene expression and foam cell formation	(85)
		To study the relationship between the phenotype-specific difference of macrophages and their ability of LDL uptake, cellular cholesterol levels, and cholesterol efflux.	(86)
		To explore whether the inhibition of bromodomain-containing protein 4 could prevent lipid accumulation in senescent macrophages	(84)
		To study the function of the RAC1 gene on regulating inflammatory cytokine secretion and lipid uptake of macrophages	(87)
		To explore the underlying mechanism of vascular inflammation effect on the foam cell formation derived from marchpane, mainly focusing on the role of NOS1 in macrophage lipid up take	(88)
	Inflammation	To investigate whether the role of TREML4 in human macrophages and the pathogenesis of atherosclerosis	(89)
		To investigate whether NOS1 could enhance the pro-inflammatory cytokine secretion by macrophages	(88)
EC	Pyroptosis	To explore the molecular mechanism of FGF21 function against atherosclerosis and the effect of FGF21 on suppressing proteins associated with pyroptosis in HUVECs	(90)
	Inflammation or/and Apoptosis	To explore whether NLRP3 activation in ECs can promote atherosclerosis development associated with diabetes	(91)
		To study how disturbed flow regulating enzymes as well as their roles in the apoptosis and inflammation	(92)
		To explore how exosome lncRNA GAS5 regulates apoptosis of HUVECs in atherosclerosis	(93)
	Cell senescence	To study whether the disturbed flow can induce HUVEC senescence and associated pathway	(94)
SMC	Phenotypic modulation	To study the oxidized lipid effect and SMC phenotype changes	(72)
		To study whether there are differences between Ox-LDL-loaded SMCs *in vitro* and *in vivo* at a genetic level.	(95)

*LPS, Lipopolysaccharide; PHACTR1, Phosphatase and Actin Regulator 1; IgE, Immunoglobulin E; NOS1, Nitric oxide synthase; lncRNA, Long non-coding RNAs; FGF21, Fibroblast growth factor 21; HUVECs, Human umbilical vein endothelial cells; GAS5, Growth Arrest Specific 5; ECs, endothelial cells; Ox-LDL, Oxidized lipoprotein*.

## Co-Culture Systems

### Direct Cell-to-Cell Interaction

Direct cell-to-cell interaction involves the culture and seeding of different cell types either on top of one another or next to each other with direct contact on TCP. This type of co-culture system has been used to study cell interaction and adhesion influenced by specific factors involved in the pathogenesis of atherosclerosis. For example, Goldschmidt-Clermont et al. developed a co-culture system consisting of vascular smooth muscle cells (VSMCs), monocytes, and macrophage colony-stimulating factor (M-CSF), to investigate macrophage activation and adhesion to VSMCs. Their results showed significant adhesion and clustering of M-CSF activated macrophages on the VSMCs and a VSMC apoptosis rate up to 60% (96). Later, Natarajan et al. expanded upon using diabetic VSMCs and the monocyte system to study diabetic condition impact on monocyte adhesion and atherosclerosis development. Through such a co-culture model, they found that monocyte binding on VSMCs was significantly higher in the presence of glucose in a dose-dependent manner, indicating diabetes may promote atherogenesis (97). Changes in VSMC phenotype due to vascular injury play a critical role in developing atherosclerotic lesions. Thus, in another study, Panitch et al. developed a hyperplastic cell co-culture model to the close mimicry of vascular injury and studied vascular injury's effect on SMC phenotype changes. For creating the injury model, a low density of ECs was seeded onto aortic smooth muscle cells (ASMCs). Utilizing this model, they showed that incomplete endothelialization resulted in switching the phenotype of ASMCs from healthy and contractile to an uncontrolled proliferative state, which plays an essential role in promoting atherosclerotic lesion development (98).

Besides that, using 2D co-culture systems, many studies have examined the correlation between chronic multi-bacterial infections, such as periodontal bacteria, and their impact on atherosclerosis development (99–102). Specifically, Yamamoto et al. created a co-culture of monocytes and human umbilical vein endothelial cells (HUVECs). They demonstrated that introducing *Porphyromonas gingivalis (P. gingivalis)*, a common bacterium in periodontal infections, to a co-culture led to a significant increase in monocyte attachment to HUVECS and inflammation (103). To further study this risk factor, Huck et al. examined *P. gingivalis* and its modulatory effects on EC apoptosis using a co-culture system composed of *P. gingivalis and* activated ECs. It was found that EC metabolic activity and cell viability significantly decreased when ECs were co-cultured with *P. gingivalis* (104). Also, establishing a co-culture system composed of *A. Actinomycetemcomitan and EC*, Lafaurie et al. showed the human coronary ECs incubated with *A. actinomycetemcomitans* generated a severer pro-inflammatory environment compared to the controls (105). These studies discussed here demonstrated that the co-culture models are crucial tools for analyzing chronic multi-bacterial infections and studying their effects on the risk factors for atherosclerosis, including inflammation, EC apoptosis, and monocyte adhesion.

Apart from mechanistic studies, 2D co-culture models have also been employed to evaluate potential treatments for atherosclerosis; nevertheless, they are not as popular as single-cell culture. One recent associated study was shown by Porrini et al., where the authors investigated the effects of polyphenols on atherosclerosis initiation using a co-culture inflammatory model composed of HUVECs, THP-1 cells, and TNF-α (106). Moreover, the Wong group reported a similar study, where the authors developed a human microvascular endothelial cell (HMVEC) and THP-1 cell co-culture system to demonstrate the remarkable efficacy of miR-146a-loaded microparticles on EC inflammation and monocyte adhesion on HMVECs (107). In another study, Li et al. also created EC and THP-1 co-culture systems to show that the newly developed peptide-based high-density lipoprotein (pHDL) could inhibit THP-1 cell adhesion and achieve a similar inhibition effect human HDL (108). Thus, these studies demonstrated an *in vitro* EC/monocyte platform for testing potential treatments for suppressing atherosclerosis initiation.

### In-direct Transwell Co-culture

Unlike direct co-culture, the indirect co-culture model utilizes transwells, providing a platform to investigate cellular responses by secretory pathways and cytokine production. The model utilizes a well plate with transwell inserts, where at least one cell type is grown on the transwell membrane filter insert or the bottom of the well of the plates.

Studies utilizing the transwell indirect co-culture model aim to explore the non-contact interactions between ECs, monocytes, and SMCs, thereby gaining a deeper understanding of atherogenesis. For instance, Natarajan et al. examined direct and indirect interactions of THP-1 cells with VSMCs using a transwell model. In this study, THP-1 cells were cultured with HVSMCs at the bottom of the well plate or in the transwell insert alone. The binding of VSMCs to THP-1 cells in the well plate led to increased Akt phosphorylation and THP-1 proliferation. In contrast, the THP-1 cells alone in the transwell insert did not show increased proliferation. This finding demonstrated that physical contact of monocytes and VSMCs might represent a critical mechanism accounting for abnormal accumulation of monocytes in the plaque, a crucial step leading to the development of atherosclerosis (109). A similar study performed by Maffia et al. demonstrated a new 2D co-culture transwell model to investigate the interaction among three key cell players in atherosclerosis development without any direct cell-cell contact. The model consisted of THP-1 in the well as well as SMCs and ECs on the underside and upper surface of the transwell insert, respectively. Findings showed that soluble factors released from ECs/THP-1 along with SMCs promoted CDH5 expression, indicating that the co-culture systems could improve the EC junction integrity (16). Similarly, Lee et al. used a transwell model to examine how the glycolaldehyde-induced advanced glycation end products (glycol-AGEs) would affect the proliferation and inflammation of SMCs. The transwell system was created by seeing SMCs in the well plate and then co-culturing HUVECs and THP-1 cells in the transwell insert. Interestingly, using this system, it was found that SMC proliferation and inflammatory cytokine production were increased upon adding glycol-AGEs in the co-culture system, while no differences were observed in the SMC-only group. Thus, this study suggested that a transwell co-culture system was of great importance for evaluating the effects of a specific compound on cell proliferation and inflammation for atherosclerosis research (110).

Various research groups have reported using transwell models to study the effects of interactions between ECs and monocytes on trans-endothelial migration, promoting atherosclerosis development. For instance, Hajishengallis et al. incorporated a transwell co-culture model to investigate monocyte adhesion and migration under chemoattractant-induced inflammatory conditions induced by P. *gingivalis* (111). For making this model, a confluent monolayer of HUVECs was first seeded in the transwell insert and cultured with THP-1 cells transfected with human CD14 and *P. gingivalis*. By utilizing such a system, substantial evidence showed that the activation of the CD11b/CD18 receptors by *P. gingivalis* increased THP-1 cell adhesion to the HUVECs and improved the THP-1 migration rate and amount through the confluent HUVEC layer to the well plate (111). In addition, to study the development of foam cells from peripheral blood mononuclear cells (PBMCs) in an atherogenesis environment, Muller et al. created a unique transwell model with HUVECs, TNF-α, and LDL in a transwell insert and PBMCs in the well plate. Through this specifically designed system, the authors found that TNF-α activated HUVEC led to a modification of LDL into Ox-LDL and resulted in the formation of foam cells derived from PBMCs (112). The study demonstrated the significance of using the transwell system to understand foam cell formation's mechanism in an early stage of atherosclerosis.

### 2D Cell Sheets

As discussed in the previous sections, many co-culture methods have been established for studies of cell-cell interactions; however, culture plate surfaces do not adequately mimic extracellular matrix (ECM) conditions, therefore impacting the cell behaviors (113). In contrast, native scaffolds with a unique avenue to overcome these limitations have been used in developing tissue-engineered 2D cell sheets (114). Taking decellularized ECM scaffolds as examples, they can provide a biocompatible and physiological mimicking environment necessary for cell adhesion, migration, proliferation, tissue morphogenesis, differentiation, and eventual homeostasis (17, 115, 116). In addition to decellularized ECM, scaffolds made by collagen and fibrin, the predominant structural proteins within the ECM, also allow cell adhesion and growth, cell-matrix interaction, and tissue function regulation, which have also been employed for cell sheet fabrication. One interesting study was conducted by the Kim group, where the authors generated cell sheets by decellularizing carotid arteries first, then using the obtained ECM from the arteries for bone-marrow cells (BMCs) seeding and differentiating the BMCs into EC and SMCs (117). In addition to carotid arteries, Choi et al. created decellularized ECM from human adipose tissue and utilized it to fabricate cell sheets with fibroblasts, SMCs, chondrocytes, and ECs (118–120). Those sheets mimic the cell components of an artery, which may serve as great tools for exploring atherosclerosis development mechanisms. Although decellularized ECM was shown to generate cell sheets in these studies, 2D models using decellularized ECM for atherosclerosis models are limited by mechanical stability, batch-to-batch variability, and ECM content qualification (121).

In addition to decellularized ECM, other scaffolds have also been used to develop cell sheets that can partially mimic artery structure. For instance, Wong et al. seeded VSMCs on degradable, tyramine-conjugated carboxymethyl cellulose and alginate hydrogel scaffold to create patterned VSMC cell sheets that could be stacked in alternating angles to mimic the native arterial medial layer (122). Kim et al. also made SMC cell sheets by seeding SMCs on dishes coated with a thermo-responsive polymer film composed of polyurethane acrylate, glycidyl methacrylate, polyethylene terephthalate with amine-terminated poly (N-isopropyl acrylamide) (PIPAAm) (123). Because of the temperature-responsive property of PIPAAm, the cell sheets could be detached by reducing the culture temperature (123). PIPAAm based approach has been widely used for cell sheet fabrication, which was discussed in the previous review (124). Nevertheless, because of the long detachment process and high cost of PIPAAm coated dishes, polystyrene has been investigated as a cost-effective alternative to fabricating the pre-vascularized SMC sheets (125). Although cell sheets have not been employed as *in vitro* models for atherosclerosis-related application, they hold great potentials for atherosclerosis research due to their layered structures with cellular components similar to the tunica intima and media of the human artery.

### 2D Microfluidic Chips

Recently, advanced *in vitro* systems, microfluidic chips, which combine micro-analysis and dynamic culture, have emerged as innovative platforms for various applications, ranging from life sciences research to drug screening and analysis (7, 8). Compared to conventional 2D systems on TCP, microfluidic chips have distinct advantages, such as enhanced sensitivity, continuous monitoring and feedback, the inclusion of flow, and continuous medium supply, making them promise candidates for conducting research associated with atherosclerosis. Generally, microfluidic devices can be classified into 2D or 3D models, dependent on the culture methods. However, in this section, we only summarize the 2D microfluidic chips (126). The 3D microfluidic chips will be discussed in the later 3D model section.

**Figure 3:**
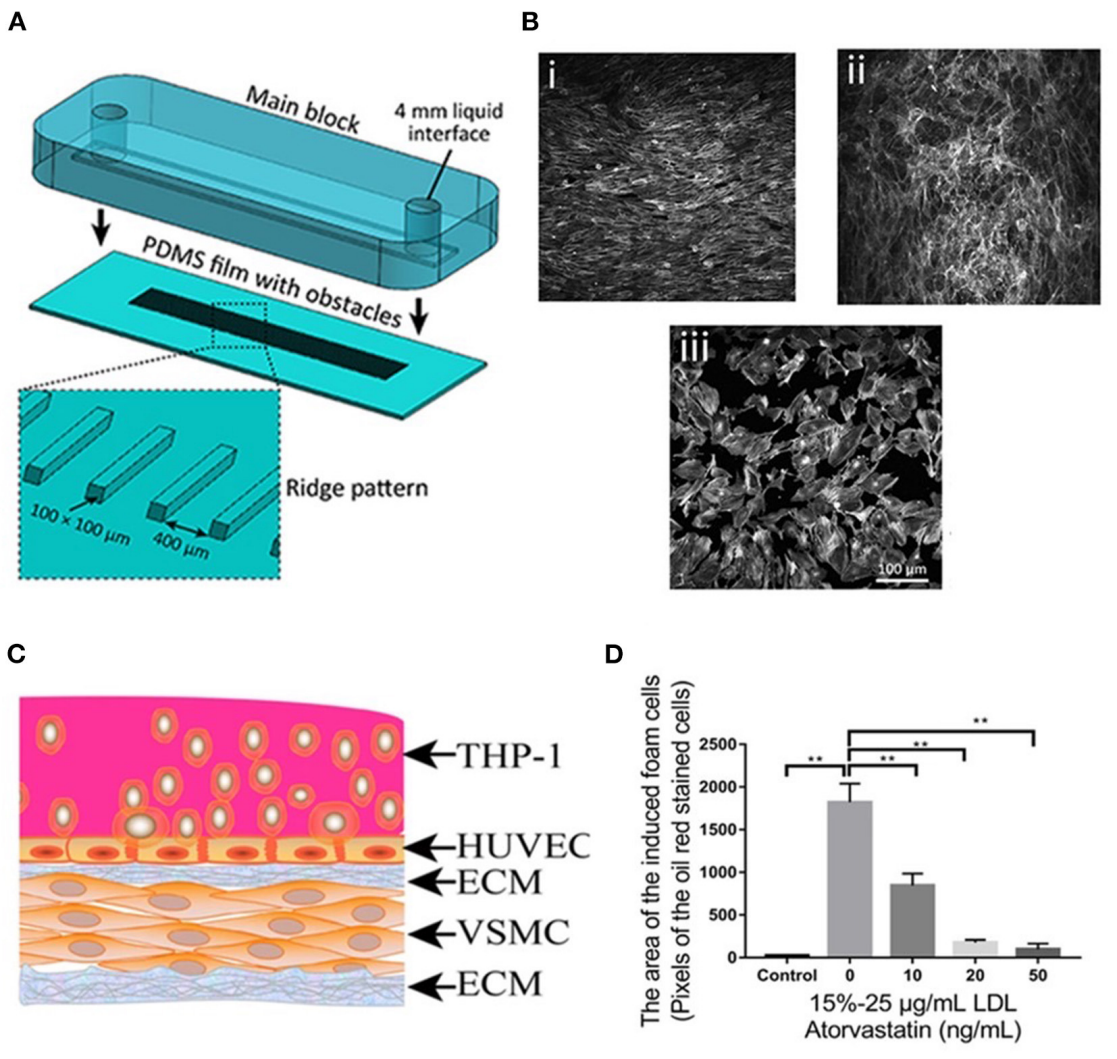
**(A)** The process of assembling the main block and the PDMS substrate with ridge obstacles, with the inset showing the zoomed-in PDMS substrate. **(B)** A confluent layer of HAECs cultured under (i) laminar flow, (ii) disturbed flow, and (iii) static condition, following which the actin cytoskeleton was labeled with Atto 565-phalloidin. **(C)** Diagram of the co-culture model. **(D)** Foam cell formation after being treated with atorvastatin and LDL at different concentrations. Adapted, with permission from (130) **(A,B)** and (131) **(C,D)**. **P < 0.01.

Endothelium plays an essential role in maintaining vessel functions, vascular integrity, and homeostasis. Dysfunctional endothelium with high permeability is regarded as one of the hallmarks of atherosclerosis initiation (127). Modeling functional endothelium *in vitro* is particularly important to improve our knowledge of atherogenesis at the molecular level. Thus, tremendous efforts have been devoted to the rational design of endothelium-on-a-chip to understand the effects of various factors on EC function. For instance, Jiang et al. developed an endothelium-on-a-chip to investigate the effects of shear stress, glucose, LDL on reactive oxidative species (ROS) production and EC function in the early atherosclerosis stage. To generate a model to closely mimic the hyperglycemia or hyperlipidemia environment in the atherosclerosis prone area, ECs were seeded in the chip to form an endothelium-like monolayer, followed by treating the EC layer with glucose or LDL under low fluid shear stress and cyclic stretch, respectively. Using this model, the authors found that the ECs seeded on the hyperlipidemia chip model showed a sharper decrease of VE-cadherin level than the ECs exposed to the same condition but cultured on the peri dish (128). This observation indicated a more pronounced cellular response from the chip model than the peri dish model, demonstrating the advantages of using chip models for atherosclerosis studies. Also, to investigate the effects of two ECM proteins, fibronectin and collagen, on endothelial inflammation, Gweon et al. created a microfluidic EC chip model by incorporating fibronectin or collagen-coated polyacrylamide hydrogel onto a chip followed by EC seeding (129). An interesting result was obtained by using this model—the ECs on fibronectin hydrogel showed a more disrupted barrier, higher permeability, and less prominent cellular elongation and orientation than the ECs on collagen-coated hydrogel upon shear stress (129). In addition, in a recent study, an endothelium-on-a-chip with specially designed ridged-shaped patterns was developed by Baratichi et al. to explore how disturbed flow affected the EC orientation, size, and nuclear shape using an endothelium-on-a-chip with specially designed ridged-shaped patterns (Figure 3A) (130). This unique design allowed the system to generate disturbed flow with low shear stress between the ridges on the chip. With the application of such system, it was revealed that, under the disturbed flow generated by the chip, EC stress fiber orientation was perpendicular to the flow, in great contrast to the ECs exposed to laminar flow or static condition, of which the fibers showed alignment along with the flow or no specific pattern, respectively (Figure 3B). Additionally, the disturbed flow was found to increase the nucleus circularity index of the cells but decrease the nucleus area compared to the laminar or static flow (130). This study demonstrated the great value of endothelium-on-a-chip for studying the hemodynamic force effect on ECs. In another study, Liu et al. endeavored to use an endothelium-on-a-chip model, and ICAM-1 modified nanoparticles for real-time evaluation of TNF-α triggered endothelium activation. This chip system included two channels separated by a semi-permeable membrane for seeding cells and activating endothelium, allowing the real-time monitoring of ICAM-1 expression on the activated endothelium by analyzing the amount of ICAM-1 antibody-modified NPs binding to the endothelium-on-a-chip. The study showed the great potential of using endothelium-on-a-chip for real-time atherosclerosis mechanism study (132).

Monocyte recruitment by dysfunctional endothelium promotes atherosclerosis progression. The design of endothelium-on-a-chip to elucidate the mechanism of monocyte-endothelium interaction has attracted increasing research interest. An example was demonstrated by Hou et al., who developed a constriction controllable endothelium-on-a-chip to investigate the monocyte attachment to the ECs stimulated by 50 or 80% constriction conditions (132). Remarkably, the authors demonstrated that the THP-1 cell attachment to the ECs was strongly dependent on constriction (132). In another study, Jeon et al. designed an endothelium-on-a-chip model to explore the interaction between THP-1 cells and ECs upon lipopolysaccharide (LPS) stimulation. Utilizing such a chip, the authors found that LPS could induce a longer migration distance of THP-1 through endothelium (133).

Along with elucidating the crucial factors regulating EC function and interaction between ECs and monocytes, the endothelium-on-a-chip has also been employed to evaluate nanoparticle behaviors in an atherosclerosis environment. For example, in one study, an endothelium-on-a-chip system was developed to evaluate the translocation of lipid-hybrid nanoparticles over dysfunctional endothelium (134). Dysfunctional endothelium was induced by treating the endothelium-on-a-chip with TNF-α and controllable shear stress. Notably, using this chip system, the authors found that the nanoparticles could translocate through the dysfunctional endothelium but were excluded by the healthy endothelium. Notably, the *in vitro* data obtained from the chip system was well-correlated with the result observed *in vivo*, indicating that the *in vitro* chip model can predict nanoparticle behavior *in vivo* (134). Likely, Jiang et al. demonstrated the combined use of endothelium-a-chip models and animal models to evaluate the potential of using platinum-NPs to treat atherosclerosis. The *in vitro* chip model results showed great consistency with the *in vivo m*odel's data, which demonstrated that the antioxidant property of platinum-NPs that scavenged hyperlipidemia induced ROS in ECs *in vitro* and decreased the expression of vascular cell adhesion protein 1 (VCAM-1) *in vivo* (128). These studies discussed here showed a good promise of the endothelium-on-a-chip system to study the nanomedicine effect on dysfunctional endothelium.

Apart from the simple chip systems only including ECs, Ding et al. established a stretchable microfluidic chip model composed of VSMC layer, HUVEC layer, foam cells, LDL, and a non-uniform stretched chip film to investigate the efficacy of atorvastatin and associated underlining molecular mechanism (Figure 3C). The stretching was induced by the manual deformation of the chip film where the cells were cultured, thus, generating an atheroprone-like microenvironment with disturbed shear stress. Using this model, the authors found that atorvastatin (50 ng/mL) significantly inhibited foam cell formation (Figure 3D), reduced ROS, and led to an up-regulation or down-regulation of crucial genes associated with atherosclerosis. More importantly, based on these data generated by utilizing the model, the authors proposed an appealing working mechanism of atorvastatin in atherosclerosis (131). This study demonstrated that endothelium-on-a-chip could not only be applied for drug evaluation but also a mechanistic investigation related to atherosclerosis development.

## *In vitro* 3D models for Atherosclerosis Studies

Traditional *in vitro* 2D platforms for atherosclerosis studies are limited to 2D, lacking physiological 3D structure observed in *in vivo* and failing to provide proper pathological compositions of human atherosclerotic plaque. In addition, 2D culture also has issues with substrate topography and stiffness (135). Therefore, the data obtained from the 2D culture system may provide misleading information regarding the safety and efficacy of the lead compound. In contrast, 3D culture, which has emerged as a helpful approach, can generate cell constructs that recapitulate the 3D structure of the organ with more naturally grown ECM, thus providing better EMC-cell and cell-cell interaction and allowing appropriate cell behaviors observed in *in vivo* (126, 136). Moreover, the 3D models can improve the predictability of therapeutic toxicity and sensitivity. It has been reported that the drug response of the 3D model is different from that of the 2D model; significantly higher drug resistance has been observed in the 3D model compared to 2D models (10). Given their unique advantages, developing *in vitro* 3D models has been of growing interest for atherosclerosis modeling and drug testing. Generally, *in vitro* 3D models can be developed by bio-fabrication, an approach that generates organized structures with biological function using living cells, cell aggregates, biomaterials, and bioactive molecules *via* bio-assembly or bio-printing followed by tissue maturation (137).

## *In vitro* 3D Spheroids

3D spheroid is three-dimensional cellular aggregates that approximately resemble a sphere, increasingly being utilized to evaluate therapeutics because of its better similarity to natural tissue than 2D culture systems. The formation of spheroid cultures involves extracellular matrix fibers with a ligand motif, such as tripeptide Arg-Gly-Asp, and their binding with integrin membrane proteins on the cell surface (138). This binding is essential as it allows the numerous cells to aggregate and facilities the binding of homophilic cadherins between cells, resulting in solid adhesion and compaction of the cell mass, leading to a spheroid formation (138). Several fabrication methods have been used to develop spheroid culture over the past several decades. One of the earliest methods used is the hanging drop method (139), in which cells aggregates form when inverting a plate with a suspension of cells. A centrifuge (140) or a spinner flask has been applied to force cells to assemble into cell aggregates to form spheroids (19, 141). Spheroids have also been produced by culturing a suspension of cells on a non-adherent substrate, forcing the cells to form spheroids (142). It should be noted that, as a result of spheroid formation, the gene expression (143, 144), metabolism (145), and cellular motility differentiation (146–148), and polarity of cells (149) within the spheroid culture were found different from that of monolayer cultures in 2D. With more accurate mimicry of structures of native tissue than traditional 2D monolayer cultures, spheroid models have been increasingly utilized for studying and modeling atherosclerosis.

As stated earlier, foam cells are differentiated macrophages that uptake lipids, the main cellular component in atherosclerotic plaque. In recent years, scientific efforts have focused on the generation of foam cell spheroid model to investigate the effects of specific compounds on foam cell formation and associated inflammation. For example, using a 3D spheroid model of foam cells, Nguyen et al. demonstrated that foam cell formation could be significantly decreased by dexamethasone (Dex) and fluocinolone acetonide (FA), but FA was more effective than Dex (150). Hydroxyl beta-cyclodextrin (HBCD) is a polysaccharide that increases cholesterol efflux and solubility. In another study, Kwan et al. used the foam cell spheroid model to evaluate the efficacy of co-delivery of HBCD and sirolimus loaded poly-*co*-lactic-*co*-glycolic acid microparticles (mc-PLGA-MPs) on foam cell formation under ultrasound stimulation (151). These studies shed light on the utility of the foam cell spherical model for atherosclerosis studies.

VSMC is another significant factor that affects arterial wall thickening, promotes atherosclerotic plaque formation, and regulates atherosclerotic plaque stability. Therefore, a study reported by Chun et al. demonstrated the effects of membrane-type 1 matrix metalloproteinase (MT1-MMP) on mouse VSMC proliferation using 3D spheroid models composed of mouse VSMCs with silenced MT1-MMP gene (152). Their results demonstrated that MT1-MMP gene, when silenced, remarkably increased the proliferation of mouse VSMCs in this 3D model; while using a 2D model, minimal effects of such gene on VSMC proliferation was observed. This study potentiated the importance of using 3D spheroid models to investigate biological processes associated with atherosclerosis other than 2D models. Furthermore, the focal adhesion kinase (FAK) has been reported to control the proliferation of VSMCs through N-Cadherin and is significant to cell adhesion. Then, Vaidyanathan et al. created a spheroid VSMC model to study the focal adhesion FAK gene and the regulation of its downstream genes, such as Rac, Rho, and Cdc42, aiming to identify potential pathways to treat neointima formation (153). By quantifying the expression of Rac and Rho in the VSMC spheroids, the authors found that FAK-Rac-N-cadherin or FAK-Rho-N-cadherin are necessary for VSMC spheroid formation, which might be considered a future target for treating atherosclerosis-related neointima formation (153).

The models demonstrated above have focused on developing an early atherosclerosis model with one type of cell component; however, it is worth noting that atherosclerosis is a chronic inflammatory disease encompassing various stages. When it comes to an advanced atherosclerosis model, Weber et al. pioneered an *in vitro* spheroid pseudo-atherosclerotic plaque composed of a spheroid core and a layer of myofibroblasts surrounding the core to emulate the late-stage atherosclerotic lesion, human fibroatheroma (15). Specifically, two types of pseudo-atherosclerotic plaque, b- and t-plaques, were developed using blood-derived myeloid cells and THP-1 cells for core fabrication. In addition to monocytes, their spheroid cores were also filled with collagen, lipid matrix with macrophages, and dendritic cells. It was found that the cell population distribution between the t-plaques and b-plaques was similar, including main components such as monocytes, macrophages, activated dendritic cells, plasmacytoid dendritic cells. Nevertheless, their components differed from human carotid plaques, primarily composed of activated dendritic cells and plasmacytoid dendritic cells (15). Moreover, native carotid plaques showed significant down-regulation of pro-inflammatory and remodeling genes than pseudo-plaques (15). Although this may be the most cohesive *in vitro* spheroid atherosclerosis model, age, sex, or genetic predisposition has not been taken into account.

## *In vitro* 3D Cell-Laden Hydrogel Constructs

Cell-laden hydrogel constructs are composed of growth factors, hydrogels, and cells, providing a 3D *in vitro* environment beneficial for studying atherosclerosis. In contrast to the 2D cell sheet fabricated by seeding cells on a 2D scaffold, the cell-laden hydrogel system is usually fabricated by embedding cells into 3D hydrogel matrices with growth factors followed by static or dynamic culture (154). Notably, cell-laden hydrogel construct can be developed with desirable geometries, sizes, and compositions, thus providing an environment allowing cells to behave similarly to *in vivo* (154). With respect to atherosclerosis applications, cell-laden hydrogel constructs implement a 3D *in vivo* mimicking model to observe cellular interactions and pathophysiology and permit lower cost, improved controllability, and higher throughput compared to animal models.

An ideal hydrogel for fabricating a cell-laden hydrogel system should be biodegradable and biocompatible with good porosity and high-water content. It also should enable cell growth, proliferation, and migration by allowing the diffusion of nutrients throughout the scaffold (155). As previously mentioned, collagen is the predominant structural protein within the ECM (20, 156). Thus, to date, the cell-laden collagen hydrogel construct has been the most widely used for elucidating the effect of a particular factor on monocyte attachment. For example, Chiu et al. created a model composed of a collagen gel, ECs, and SMCs to study the influence of SMCs on inflammation and monocyte adhesion for atherosclerosis development (157). The SMC-laden collagen hydrogel was fabricated by embedding SMCs within the collagen hydrogel and seeding an EC monolayer over the SMC-laden hydrogel. Additionally, in another study, the SMC-laden collagen hydrogel was utilized to elucidate the roles of SMCs and flow in leukocyte adhesion and transmigration (157). In addition, due to the ability to mimic vessel intima-media structure mimicking, EC-seeded SMC-laden collagen-based hydrogel construct was used to model earthy atherosclerosis by the Hou group in a recent study. The authors induced EC dysfunction and SMC migration by treating the cell-hydrogel construct with IL-1β, TNFα and Ox-LDL. Notably, the SMC migration into the EC layer could be easily quantified using such a system, which cannot be achieved through a traditional transwell assay. Moreover, the potential of this system as a drug screening tool was demonstrated by the atheroprotective effect of vitamin D, and metformin was observed when tested using this model (158). However, the main limitation of this study is that the EC layer is not monolayer and is as thick as the SMC layer, and lacks tunica adventitia. In another case, the Teo group manipulated the monocyte-laden collagen hydrogel construct with low or high densities to resemble the early or late-stage atheroma atherosclerotic tissues to study the ECM (collagen) effects on macrophage behaviors in these two environments. To generate the construct, THP-1 cells were first embedded within the collagen hydrogel with Ox-LDL, then differentiated into macrophages and activated into pro- (M1) and anti-inflammatory (M2) phenotypes. By detecting the inflammatory cytokines produced by the model, the authors found that M1 macrophages, M2 macrophages, and THP-1 monocytes showed different responses in high and low tissue density hydrogel construct (159).

Besides collagen hydrogels, a fibrin gel-based model was used to model early atherosclerosis by the Vahl group. Briefly, SMCs were encapsulated into the fibrin gel first, and ECs were seeded onto the SMC-laden fibrin gel to form an EC seeded SMC-laden fibrin construct; then, lipoproteins and monocytes were added to the culture to induce atherogenesis and foam cell formation. Additionally, this model was used to study atherosclerosis development for up to 6 weeks, indicating its long-term stability. This study demonstrated an autologous *in vitro* vascular model for studying the development of early atherosclerotic lesions (160). Similarly, by using 3D engineered SMC-fibrin construct of a specific geometry, Vogel et al. discovered that the balance between metalloproteinase (MMP) and their inhibitors are flow-dependent—high shear stress could protect the *de novo* ECM, whereas low shear stress would cause SMC proteolytic activity leading to more collagen, less elastin, and shifted SMC phenotype (161). This study demonstrated the potential of using cell hydrogel construct for evaluating hemodynamic force effect on cells associated with atherosclerosis development.

## *In vitro* 3D Vessel Based Systems

### Tissue-Engineered Blood Vessels (TEBVs)

Over the past decade, significant advancements in tissue engineering, regenerative medicine, biomaterials, and cell biology enabled the fabrication of tissue-engineered blood vessels (TEBVs) as vascular grafts for treating atherosclerosis. Until recently, TEBVs have emerged as valuable models for studying atherogenesis or developed into *in vitro* atherosclerosis platforms that replicate the key features of atherosclerosis, offering an alternative to 2D and animal models for atherosclerosis and associated therapy studies.

In the case of TEBV fabrication, TEBVs have been developed by seeding vascular cells in biodegradable polymeric scaffolds. For instance, Arai et al. developed single-layered TEBV by seeding fibronectin and gelatin-coated mouse smooth muscle cells on poly-(l-lactide-co-ε-caprolactone) (PLCL) scaffold followed by maturing tissue in a perfusion system. The resultant TEBVs achieved similar mechanical properties to that of native arteries. Similarly, Lissy et al. created 2-layered TEBVs by seeding ECs and SMCs on PCL conduit with controllable wall thickness and shear stress (162). In addition to the seeding strategy, cell sheet technology has also been used as an alternative approach. For instance, single-layered TEBVs were created by the Germain group by rolling the fibroblast cell sheets into a vessel-like structure. Then, the cell sheets were fabricated by culturing fibroblasts on TCP for 1 month. With the results from single-layered TEBV, the same group also developed a multi-layered TEBV using decellularizing a single-layered TEBV followed by seeding SMCs and ECs in the decellularized TEBV and maturing the TEBV in a bioreactor (163). Besides this, an innovative strategy was unrevealed by Rolle et al. recently, where the authors developed spatially controlled TEBV by fusing SMC ring units into a vessel structure with heterogeneous compositions similar to those observed in intimal hyperplasia or atherosclerosis. In particular, the human aortic SMCs (hAoSMC) ring units were self-assembled structures by culturing hAoSMCs in agarose molds. Then, the TEBV was created by threading the hAoSMC ring units on a mandrel and then culturing the mandrel with ring units in a static condition, followed by a dynamic environment using a bioreactor (164). Although some TEBVs have been generated by seeding cells on 2D scaffolds followed by rolling and maturing of the vessel structure, we still discuss them in the 3D section as those TEBVs provide a 3D vessel shape.

For atherosclerosis-associated applications, 2-layered TEBVs were developed by the Truskey group to demonstrate that PCSK9 might affect atherogenesis, independent of LDL. Apart from PCSK9, the same group also explored the effect of oxidative stress on inflammation and senescence on vascular cells using a 2-layered TEBV composed of endothelium and fibroblast layers. The data generated from the model suggested that oxidative stress promoted atherosclerosis by increasing vascular cell inflammation (165–167). Likely, Chen et al. developed a 2-layered EC-SMC TEBV. Using an imaging chamber, the authors observed the real-time dynamic process of leukocyte recruitment and penetration through the endothelium into the intima using the developed TEBV. This study was the first demonstration of the real-time monitor for *in vitro* pathogenesis, which enable a more thorough investigation of drug effects on cellular behavior in a micro-physiological system for understanding atherosclerosis pathogenesis (168).

Although significant early attempts were made with the design of TEBVs without curvature, recently, the interest in developing branched TEBVs for disease studying has been grown, attributed to the fact that different vascular geometries leading to varied flow patterns may significantly affect atherogenesis. One typical example was demonstrated by the Leong group, where the authors constructed a branched TEBV to study the flow pattern effect on atherogenesis. They found that the athero-prone region (branched side outlets) demonstrated more monocyte adhesion than other areas (169). In another study, Cardinal et al. developed an angulated TEBV that mimics the bent human vessel to study the stent effects on atherogenesis in athero-prone regions (170). The TEBV was developed by seeding HUVECs on the expanded polytetrafluoroethylene tubular scaffold with bent geometry. The authors found a significantly reduced endothelialization on the stented TEBV compared with the un-stented control (170). These studies demonstrated that an *in vitro* TEBV platform with controllable geometry could mimic the athero-prone condition of the artery, which is well-suited for future intravascular device evaluation.

**Figure 4:**
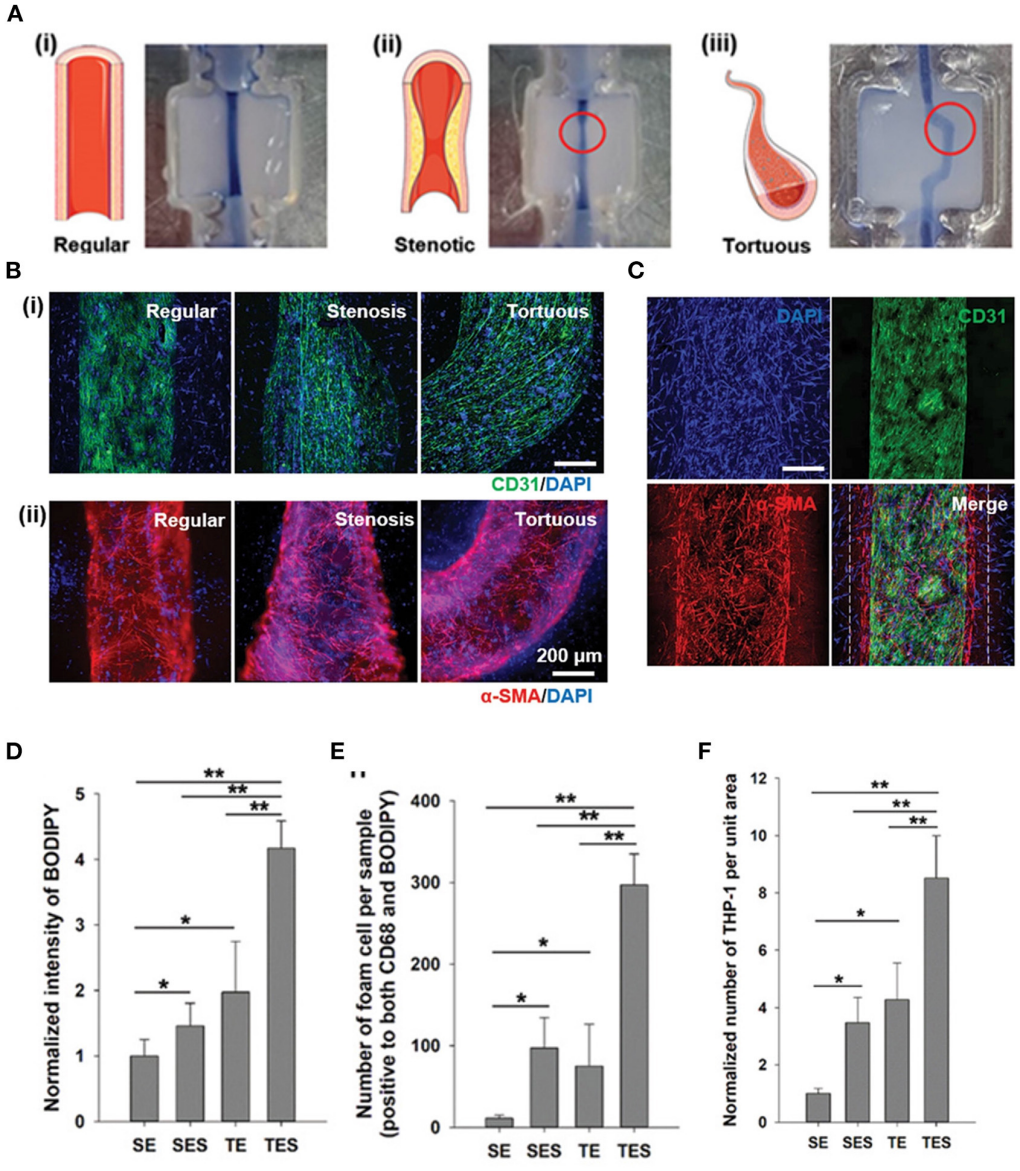
Construction of a novel *in vitro* atherosclerotic model from geometry-tunable artery equivalents engineered *via* in-bath coaxial cell printing. **(A)** By programming the printing path and moving speed, triple-layer arterial constructs with controlled geometries were achieved, including (i) regular straight, (ii) stenotic, and (iii) tortuous models. **(B)** (i) Confluent endothelium and (ii) dense smooth muscle tissues were generated in the constructed artery equivalents on day 7, regardless of the designed geometries (Scale: 200 μm). **(C)** The distributions of a monolayer endothelium surrounded by compartmentalized smooth muscle cells and fibroblasts (white dashes) are distinguishable **(D)** Quantification of LDL accumulation, **(E)** foam cells, and **(F)** adhered THP-1 cells. SE: steady-flow model containing only ECs; SEC: steady-flow model containing ECs and SMCs/fibroblasts; TE: turbulent-flow model containing only ECs; TES: turbulent-flow model containing EC and SMS/fibroblasts. Adapted, with permission from (171). *P < 0.05; **P < 0.005.

Compared to TEBV, TEBVs with atherosclerotic features, later referring as diseased TEBVs, have brought more enthusiasm to the field. However, due to the complexity of atherosclerotic plaque, only a few studies have developed diseased TEBVs with some key features of atherosclerosis. One representative example was demonstrated by Hoerstrup et al., who fabricated a 2-layered diseased TEBV by seeding vascular cells on a biodegradable tubular scaffold and adding LDL and inflammatory cells under high/low shear stress. The diseased TEBV platform demonstrated early atherosclerosis features such as monocyte attachment and LDL (21). Besides 2-layered diseased TEBV, Truskey et al. endeavored to fabricate a 3-layered TEBV (ECs, SMCs, and dermal fibroblast, from inside to outside) and induced early atherosclerosis using LDL with/without TNF-α (18). Their method maintained the vascular cell phenotype and early atherosclerosis symptoms in the TEBV, including endothelial activation, vasoactivity, monocyte accumulation, foam cell formation, and macrophage polarization. Moreover, they used this model to test the effects of LDL, lovastatin, and P2Y_11_ inhibitor (NF157) on disease progression and found that lovastatin can block the altered vasoactivity and NO production induced by eLDL and TNF-α (18). Therefore, this diseased TEBV could be used to study specific vascular functions that might be challenging to evaluate *in vivo*. Similarly, in another study, Cho et al. fabricated atherosclerotic three-layered vascular construct conduits with tunable geometry (stenosis and tortuous structure), a monolayer of the confluent endothelium, and condensed SMC layers using cell printing technology (Figures 4A–C). Significantly, the turbulent flow in the TEBVs with stenosis and tortuous structure and co-culture of SMCs and ECs led to higher endothelial dysfunction LDL accumulation (Figure 4D), foam cell formation (Figure 4E), and THP-1 cell recruitment (Figure 4F), hallmarks of early atherosclerosis compared to other conditions. These results indicated the importance of athero-prone vascular structure and the co-existence of vascular cell types to generate atherogenesis in the model. Remarkably, the authors showed the significant role of TEBV in atherosclerosis drug testing by demonstrating that atorvastatin efficacy for atherosclerosis was observed in the TEBV, including endothelial dysfunction, monocyte recruitment, LDL oxidization, and uptake, and improvement of free cholesterol efflux. Hence, this study substantiated the TEBV as a promising tool for biomedical applications, including pathological study and novel drug identification and evaluation (171).

### Vessel-on-a-Chip

Vessel-on-a-chip is one type of organ-on-a-chip (OOC) system, which has aroused significant interest in researchers working in the field of atherosclerosis studies. Notably, an OOC system is a biomimetic *in vitro* microfluidic platform developed by combining cell biology, microfluidic technology, biomaterial science, and tissue engineering. Typically, an OCC system possesses an engineered architecture built in a chamber of a micron-sized electron fluidic chip, recapitulating the micro-physiological environment and architectures of functional human organs (172, 173). The chamber can be connected to a pump with a controlled flow rate and shear stress (174). In other cases, critical features of certain human diseases in a specific tissue can be induced in the OCC for modeling disease (175). Compared to traditional 2D static cultures, OOC systems provide a dynamic environment with more accurate vascular physiology, morphology, and response. Furthermore, high throughput capacity is a compelling feature of OOC systems in comparison to animal models, which is important for reducing the R&D cost (176). Therefore, OOC systems, particularly vessel-on-a-chip, have emerged as cost-effective platforms to fundamentally study biochemical and metabolic processes, investigate cellular responses during atherogenesis, and evaluate the therapeutic efficacy and safety for atherosclerosis (177). Hence, in the following section, we overview the recent progress in this field.

Vessel-on-a-chip comprises a vessel-like structure on a chip with or without disease features. Vessel-on-a-chip systems have been fabricated by seeding cells on a fiber scaffold. For instance, to tackle the issues commonly observed in vessels developed in static conditions, such as endothelium shedding and irregular orientation, the Li group fabricated a chip by seeding ECs on a highly oriented electrospun poly(ε-caprolactone fibers) scaffold. The ECs grown on the chip showed improved endothelialization under perfusion and achieved alignment under a flow similar to natural vessel endothelium (178). Besides direct seeding cells on a scaffold, vessel-on-a-chip has also been made by fabricating cell-laden constructs and seeding cells on the hybrid constructs. For example, Khademhosseini et al. fabricated by constructing fibroblast and SMC laden-gelatin methacryloyl gel, followed by seeding ECs on the SMC-laden construct through perfusion (179). After 3 days of perfusion, the vessel showed a confluent endothelium layer, with significantly better barrier function than the vessel without an EC layer (179). This vessel-on-a-chip system is highly applicable for drug safety screening because of its three-layered structure and perfusion system. In another study, Li et al. reported their blood-vessel-on-chip with controllable vessel structures (straight, wavy, or helical) to mimic various blood vessel physiological environments. First, the authors fabricated hollow microfiber with a straight, wavy, or helical structure by controlling the flow rate, injection device radius, and fluid composition of each channel. Then, a fully covered endothelium layer was achieved by seeding HUVECs in the inner surface of collagen and alginate-coated hollow microfibers. Additionally, a proof-of-concept test was conducted to show that this cell-laden microfiber could be fabricated into a vessel-on-a-chip (180).

Although the progress of significance toward the development of vessel-on-a-chip systems has been made, most studies have focused on the generation of non-diseased vessel-on-a-chip with limited applications, with only one study demonstrating the development of atherosclerotic vessel-on-a-chip. In this study, Hoerstrup et al. induced atherosclerosis on a chip with tissue-engineered arteries (hiTEV) made from cells derived from human-induced pluripotent stem cells (hiPSCs) (181). To generate the hiTEV, the authors firstly induced SMC, EC, and macrophage-derived from hiPSCs and showed that the hiPSCs-derived SMCs and ECs could express SMC contractile phenotype markers and endothelial phenotype markers. Then, the authors fabricated two-layered hiTEV by seeding hiPSC-derived SMCs in the biodegradable tubular-shaped conduits carrying fibrin scaffold and cultured, followed by seeding the hiPSC-derived ECs in the inner lumen of the conduits on a microfluidic chip. It was found that the matured hiTEV was composed of endothelium and SMC layer with good expressions of EC and SMC phenotype markers, respectively. Furthermore, plaque-like structures were induced in the hiTEV after the vessel was treated with hiPSC derived macrophages and LDL. The authors also found that populations of dendritic cells for conducting antigen-presenting, ECs, and macrophages from the tissue-engineered plaque showed resemblance to those found in native plaque. Notably, the transcription expression of ECM assembly and remodeling of tissue-engineered plaques also displayed similarity to native plaques (181). Although this model emulated the native plaque closely, some limitations and drawbacks existed. For instance, hiPSC derived cells did not possess aged but fetal-like cell phenotype, while atherosclerosis occurred in young and older adults. Secondly, T and B cells play essential roles in atherosclerosis development; however, T and B cells were not included in the developed model. In addition, the applications of such a system were not demonstrated in the study.

**Figure 5:**
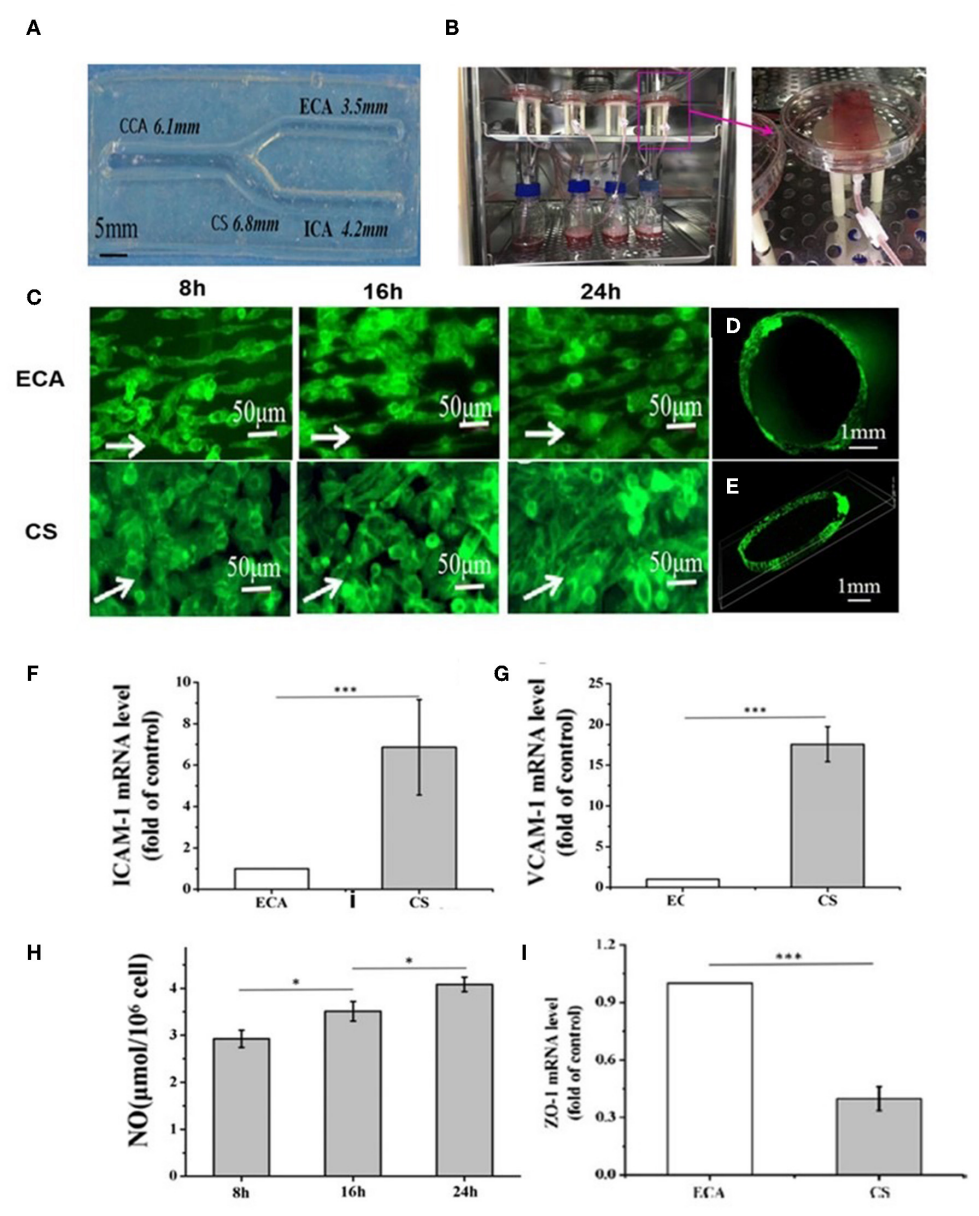
**(A)** Gelatin-based carotid artery model. **(B)** Actual assembled carotid artery system. **(C)** ECs morphology in Region ECA after dynamic experiment 8, 16, and 24 h or ECs in Region ECA. ECs morphology in Region CS after dynamic experiment 8, 16, and 24 h. **(D,E)** Distribution of ECs in cross-section of Region ECA (The white arrows point in the direction of flow). **(F–I)** The expression of ICAM-1 **(F)** and VCAM-1 **(G)** was studied in the Regions ECA and CS after 24-h perfusion experiment; **(H)** The vasoactive substances NO; **(I)** Expression of ZO-1 was studied in the Regions ECA and CS after 24 h perfusion. Adapted, with permission from (183). *P < 0.05; ***P < 0.001.

Vessel-on-a-chip systems have been applied to investigate the biological process associated with atherosclerosis and evaluate the safety of specific compounds. For example, Joore et al. designed tubular vessels-on-a-chip to study monocyte adhesion to endothelium. The tubular vessels were fabricated by seeding human coronary artery ECs into collagen gel and culturing the cell-gel complex using perfused flow. The authors showed that after being treated with TNF-α, the vessels expressed significant ICAM-1 proteins and recruited significant monocytes. Moreover, using this model, the authors found that aerosol extract was far less toxic than cigarettes exact to the endothelium by showing that aerosol extract induced significantly lower expression of ICAM-1 and monocyte adhesion (182). In another study, Li et al. reported the generation of an endothelial carotid artery model using EC and gelatin (183). More importantly, this study demonstrated the first tuning fork-shaped artery (Figure 5A), which was composed of four parts able to mimic the common carotid artery (CCA), internal carotid artery (ICA), external carotid artery (ECA), and carotid sinus (CS), respectively (Figure 5A). In addition, a perfusion loop consisted of a medium, and a pump was used to generate a flow for this model (Figure 5B). Interestingly, when the flow was applied, the laminar flow in the CCA region changed into a disturbed flow with lower WSS in CS regions because of its curvature structure. However, when the flow reached the ICA and ECA regions, it transformed into laminar flow with higher WSS. Due to the chip's unique design leading to various flow conditions in different chip areas, the authors were able to study the hemodynamics effect, such as wall shear stress (WSS), on ECs. Remarkably, they found that the ECs in the chip showed different responses to the flow changes. In the ECA region, the EC becomes elongated and aligned following the flow direction, whereas, in the region of CS, the ECs demonstrated a round shape and are more disorganized (Figure 5C). In addition, an endothelialized monolayer was found to form in the region of ECA (Figures 5D,E). Moreover, the expression of ICAM-1 and VCAM-1 in the CS region was more significant than that in the ECA region (Figures 5F,G). In addition, the WSS was found to increase the nitric oxide production in the system with time (Figure 5H). Also, a decreased expression of ZO-1, the primary EC tight junction marker, was observed in CS regions compared to the ECA region (Figure 5I) (183). This study demonstrated an example of creating and using a vessel-on-a-chip of unique characteristics to investigate the hemodynamic force effect on crucial cellular components associated with atherogenesis, which is not feasible in most other *in vitro* systems that depend on static culture.

## Conclusion and Future Perspectives

A considerable number of *in vitro* models have been developed as potential platforms for studying atherosclerosis mechanisms and exploring new treatment, ranging from a traditional 2D single-cell culture system on TCP (Tables 1, 2) to advanced 3D TEBVs and vessel-on-a-chip models (Table 3). The advantage of using *in vitro* systems for drug evaluation is that the disease indicators, risk levels, and the determination of efficacy and toxicity of specific drug treatment can be studied with reduced cost, time, and resources before advancing to complicated *in vivo* models or clinical studies. This review discusses each system and associated studies in detail and summarizes the advantages, limitations, and future perspectives of each *in vitro* system (Table 4). Although, by far, the established *in vitro* models are not yet capable of fully modeling human atherosclerosis, these models, particularly the 3D models, show excellent potential for improving atherosclerosis research and revolutionizing the drug development process.

**Table 3:** Summarization of the models, cell types, and biological processes studied using *in vitro* 2D culture systems and *in vitro* 3D systems covered in the current review.

**Model**	**Cell types**	**Model function (biological process)**	**References**
Direct cell-to-cell Interaction (2D)	VSMCs, monocytes, ECs	To study the effect of diseased conditions (M-CSF, diabetic, or vascular injury) on atherosclerotic development, including macrophage activation/adhesion to VSMC, or SMC phenotype switch	(96–98)
	Monocytes, HUVECs, bacterium	To study the impact of bacterial infection on atherosclerosis development (inflammation and EC apoptosis)	(99–105)
	ECs, THP-1s	To evaluate potential atherosclerosis treatment	(106–108)
In-direct Transwell Co-culture (2D)	SMCs, THP-1s	To study the significance of physical contact between SMC and monocyte for atherosclerosis development	(109)
	SMCs, ECs, THP-1s	To study the interactions between different cell types and their effects on atherogenesis.	(16, 110–112)
Cell Sheet (2D) (Decellularized ECM)	ECs and SMCs differentiated from BMCs; fibroblast, SMCs, ECs, chondrocytes	To mimic the native ECM component to improve vascular cell spreading and proliferation.	(117–121)
Cell Sheet (Polymer Scaffold)	SMCs	To partially mimic arterial structure	(122–124)
Microfluidic Chip (2D)	ECs (endothelium-on-a-chip), THP-1s	To observe endothelial response, inflammation, and interaction with monocytes during atherosclerosis.	(127–130, 132, 133)
		To evaluate the effect of nanomedicine on dysfunctional endothelium	(128, 134)
	Multi-layer including SMCs, ECs, and foam cells	To study the mechanism of atorvastatin under the atherosclerotic condition	(131)
Spheroid (3D)	Foam cells	To evaluate therapeutic effects on atherosclerosis	(150, 151)
	SMCs	To study SMC remodeling during atherosclerosis	(152, 153)
	Myeloid cells, THP-1s, macrophage, dendritic, myofibroblasts	To emulate late-stage fibroatheroma	(15)
Cell-laden Hydrogel Construct (3D)	ECs, SMCs, monocytes neutrophil in collagen gel	To mimic early atherosclerosis and study the effect of SMC on monocyte adhesion	(157, 158)
	THP-1s in collagen gel	To study ECM effect on macrophage behavior under early and late atherosclerosis	(159)
	ECs, SMCs, monocytes	To study early atherosclerosis development	(160, 161)
Tissue-engineered Blood Vessel (3D)	ECs, fibroblasts, or SMCs (2-layered vessels)	To study the mechanism of atherogenesis or drug screening	(165–167)
	ECs, monocytes (branched geometry)	To study the endothelial behavior in the athero-prone region	(169, 170)
	ECs, SMCs, monocytes, fibroblasts	To mimic key early atherosclerotic plaque features for mechanism study or drug screening	(18, 21, 171)
Vessel-on-a-chip (3D)	ECs, SMCs	To mimic the natural vascular features on a chip with controlled geometry	(178–180)
	ECs, SMCs, and macrophages	To emulate atherosclerotic plaque on a chip	(181–183)

**Table 4:** Summarize the features, advantages, application, challenges, and future directions of models discussed in the review.

**Model**	**Features**	**Advantages**	**Applications**	**Challenges**	**Directions**
Single-cell Systems (2D)	Seeding only one cell type in a tissue culture plate (TCP)	- High availability - Easy to make - Cost-effective - High reproducibility - High throughput	- Evaluation of drug and drug delivery system - Mechanistic studies	- Fail to mimic the native plaque composition and vascular structure	- Creating a co-culture system
Direct Co-culture (2D)	Direct cell-to-cell seeding of multicell types in a TCP		- Study of cell-cell interaction and adhesion	- Difficulty mimicking native physiological structures and proper development of cellular interactions with ECM	- Using ECM - mimicking scaffold
In-direct Transwell Co-culture (2D)	Cells seeded in a TCP and trans-well inserts		- Study of cellular responses *via* secretory pathways and cytokine production		- Improving cell attachment - Using ECM mimicking scaffold
Cell Sheet (2D)	Layered structure seeded on the 2D scaffolds or no scaffolds	- Better mimicking the vascular wall structure than other 2D systems - Relatively easy to fabricate	- Potential for therapeutic evaluation - Potential for studying cell-cell interaction	- Prone to spontaneous shrinkage or contraction - Lacking adequate mechanical properties - More expensive than TCP based systems	- Increasing mechanical property through other types of scaffolds - Exploration of applications of such systems
Microfluidic Chip (2D)	Endothelium seeded on a chip with a flow	- Micro-analysis - Providing continuous monitoring and medium supply - Enhanced sensitivity - Dynamic culture - Relatively easy to make	- Mechanistic studies - Nanomedicine evaluation - Allowing real-time imaging	- Require additional types of equipment, such as pumps, tubing, and connectors - Not adequately model 3D native environments - Costly	- Using ECM mimicking scaffold - Using multiple vascular cells observed in the plaque
Spheroid (3D)	Cellular aggregates to provide 3D structures	- Providing spherical structures - Advanced plaque	- Mechanistic studies	- Limited capability or function in comparison to native tissue - Failing to provide the layered vascular structure - Low quantity of spheroids	- Large production of this model
Cell-laden Hydrogel Construct (3D)	Cells embedded within hydrogel scaffolds	- Ability to provide an ECM mimicking environment provided by the scaffold - relatively easy to make	- Mechanistic studies	- Difficult to reproduce - Poor mechanical properties	- Increase mechanical properties and reproducibility - Controlling the properties of hydrogels - Using these systems for various applications
Tissue-engineered Blood Vessel (3D)	Models reproducing native vessel structure and size with or without disease features	- Allowing controlled stimuli - Providing partially vessel-like structure - Dynamic culture - Providing similar size to that of native vessels	- Drug evaluation - Mechanistic studies - Potential for medical device evaluation - Allowing real-time imaging	- Majority are not developed using arterial cells - Lacking the fibroblast layer - Challenging to have a monolayer of endothelium - Failing to induce advanced atherosclerotic plaques - Expensive and difficult to reproduce - Time-consuming	- Producing three-layered vessel - Using arterial cells or stem cells - Incorporating T and B cells in the system - Large scale production for pharmaceutical use - Improving reproducibility - Providing advanced plaque
Vessel-on-a-chip (3D)	Models reproducing vessel structures with or without disease feature on a micro-sized chip	- Allowing controlled stimuli and real-time imaging - Providing partially vessel-like structure - Dynamic culture	- Applicable for drug screening - Mechanistic studies - Allowing real-time imaging	- Expensive and difficult to reproduce - Additional equipment to create a dynamic microenvironment - Lacking three-layered structures - Time-consuming - Inability to provide actual size vessel for medical device evaluation - Limited to drug screening	

Due to the ease of creation, traditional *in vitro* 2D models, including single-cell culture and direct or indirect co-culture models on TCP, have been widely used to evaluate drug delivery systems and conduct a mechanistic investigation for atherosclerosis. Still, these models have some limitations in the aspects related to closely resembling physiological structures *in vivo* and promoting adequate development of ECM interactions, thus leading to improper regulation of cell signaling and behaviors and making it challenging to translate data to animal and human studies. In addition to these problems, indirect culture systems also suffer a notable drawback of insufficient cell attachment to the surface of trans wells.

Taking advantage of cell sheet engineering, a more advanced *in vitro* 2D cell sheet model has been developed to mimic the layered structure of the artery wall. The 2D cell sheets include EC, SMC single layer, or EC-SMC bilayer sheets, showing great potential for evaluating drug toxicity and developing new atherosclerosis models. However, 2D cell sheets, mainly made from a scaffold-free approach, suffer drawbacks of low mechanical property and spontaneous contraction and shrinkage. In addition to cell sheet models, with the recent advancement of microfluidic technology, endothelium-on-a-chip has been created to provide an improved *in vitro* environment to study the mechanism associated with endothelial dysfunction and monocyte attachment. Even though substantial evidence has shown that endothelium-on-a-chip systems had brought new insights into atherogenesis, such a system by far can only be applied to study biological processes in the early atherosclerosis stage. However, atherosclerosis is a complicated process, sequentially transiting from early to the advanced stage as inflammation increases, and only advanced atherosclerosis may drive severe cardiovascular disease. Thus, it is imperative to develop advanced-stage atherosclerosis models to facilitate the development of therapeutic options with improved efficacy for treating late-stage atherosclerosis.

Thanks to the advancement in tissue engineering and microfluidic technologies and the innovation of biomaterials, the fabrication of 3D *in vitro* models that recapitulate the physiological architecture and provide a pathological environment better than 2D models has become a reality. 3D *in vitro* models discussed in the review include spheroid, cell-laden hydrogel constructs, TEBV, and vessel-on-a-chip. Although the spheroid system has become a new breakout for cancer drug screening, to date, the development of the spheroid model for atherosclerosis is still in its infancy, and its application is limited. This is possibly due to its limited capability to faithfully mimic the layered structures of an atherosclerotic vessel. The most impressive spheroid system developed so far can only replicate the features of atherosclerotic plaque, not the vessel wall structure. In addition to the spheroid models, cell-laden hydrogel models have also been created for atherosclerosis studies. In contrast to the spheroid model, the distinctive advantage of the cell-laden hydrogel system is its ability to recapitulate layered structures similar to the human vessel wall. In comparison to the 2D cell sheets, the cells embedded in the layered cell-laden hydrogel behave more closely to those observed *in vivo*. Among the cell-laden hydrogel systems, collagen and fibrin-based cell-laden hydrogels are the most widely used models due to their ECM mimicking property. Although significant progress in developing cell-laden collagen and fibrin hydrogel models is highly encouraging, these systems still have drawbacks of poor reproducibility issues, lack of homogeneity, and insufficient mechanical properties. It is, therefore, worth exploring a new method to improve the reproducibility and homogeneity of cell-laden hydrogel constructs in the future. In addition, the incorporation of cell-laden collagen into microfluidic devices to generate more advanced atherosclerosis models can also be an exciting future direction. Besides, future focus can move toward the development of a cell-laden hydrogel model with improved disease features using hydrogel scaffolds made from other biomaterials, such as alginate, chitosan, hyaluronic acid, gelatin, or poly(ethylene glycol), to overcome current limitations.

With respect to *in vitro* 3D models, TEBVs and vessel-on-a-chip systems are the most prominent systems with the greatest complexities and physiological relevance, which can reproduce vessel structures and provide a disease-like spatial environment with controlled mechanical stimuli. In addition, both systems empower potent tools allowing for real-time imaging and investigating multifaceted biological processes and mechanisms contributing to atherosclerosis. However, different from vessel-on-a-chip, which is limited to drug screening, TEBVs can be fabricated to possess the actual vessel size, thereby offering an opportunity to evaluate medical devices. Although the recent achievement in TEBV and vessel-on-a-chip systems is highly encouraging and has significantly contributed to atherosclerosis studies, some limitations remain. For instance, (1) vessels in some studies were made with the mouse, dermal, or venous cells instead of human inflammatory and arterial cells; (2) most of the vessels developed were single or double-layered vessels, lacking the fibroblast layer; (3) it is still challenging to create vessel structure composed of a complete monolayer of the endothelium in vessels; (4) no studies have explored an approach for the induction of advanced atherosclerotic plaques. Therefore, future studies may focus on several vital perspectives, such as how to (1) fabricate a 3-layered vessel wall using human artery cells and (2) how to create advanced atherosclerotic features including calcification necrotic core and thrombosis in the vessels. Such advanced systems will significantly impact the development of new therapeutics for treating late atherosclerosis. In addition, with the substantial improvement in stem cell technology, future research can also explore whether the incorporation of hiPSC isolated from the patient will create a system faithfully mimicking atherosclerosis for personalized precision medicine. It is also important to address whether including immune cells (T and B cells) would generate an improved disease system. Besides that, it is imperative to explore how to produce TEBV and vessel-on-a-chip models on a large scale with excellent reproducibility. In addition, how to achieve commercialization and translation of these systems for medical end-users remain to be achieved. Moreover, the standardization of the manufacturing process of those models still needs to be performed in the near future. Lastly, in addition to the use of drugs as a therapeutic approach for atherosclerosis, medical devices such as drug-eluting stents (DESs) and drug-coated balloons (DCBs) have also been developed to treat in-stent restenosis associated with atherosclerosis. However, current commercially available DES and DCBs are found to have their issues; thus, a new coating design for improving the performance of these medical devices is needed. As aforementioned, most *in vitro* models have been developed for drug screening, with no study reporting *in vitro* systems suitable for assessing new coating designs for medical devices. Hence, developing powerful *in vitro* models for the effective evaluation of medical devices with a novel coating material is vital and should be regarded as a future research focus.

## Author Contributions

JC and XZ designed and outlined the article and primarily revised the draft. TL and YC drafted as well as JC edited the pathology of atherosclerosis section. RM, SM, and JS drafted as well as JC and PH edited the *in vitro* 2D model section. JC, XZ, TL, and MG drafted as well as JC and XZ edited the *in vitro* 3D model section. JC drafted the introduction and conclusion section. BB, GQ, Y-sY, and HJ provided suggestions for article writing and figure selection. SM, MG, and JC formatted references. H-WJ conceived, supervised, and reviewed outline and article writing. All the authors have reviewed and approved the manuscript for submission.

## Funding

The authors gratefully acknowledge the financial support from the National Institutes of Health (1R01HL125391 to H-WJ), Alabama Research and Development Enhancement Fund (1ARDEF22 09 to H-WJ), American Heart Association (18POST34080260 to JC and 20PRE35210599 to XZ), National Institutes of Health (1R01HL150887 to Y-sY), and National Research Foundation of Korea (NRF) funded by the Korea government (MSIT) (Nos. 2020R1A2C3003784 and 2020M3A9I4038454 to Y-sY).

## Conflict of Interest

RM, JS, PH, BB, and H-WJ were employed by the company Endomimetics, LLC. The remaining authors declare that the research was conducted in the absence of any commercial or financial relationships that could be construed as a potential conflict of interest.

## Publisher's Note

All claims expressed in this article are solely those of the authors and do not necessarily represent those of their affiliated organizations, or those of the publisher, the editors and the reviewers. Any product that may be evaluated in this article, or claim that may be made by its manufacturer, is not guaranteed or endorsed by the publisher.
